# Genetic Variations of NLR family genes in Behcet’s Disease

**DOI:** 10.1038/srep20098

**Published:** 2016-02-01

**Authors:** Lin Li, Hongsong Yu, Yanni Jiang, Bolin Deng, Lin Bai, Aize Kijlstra, Peizeng Yang

**Affiliations:** 1 The First Affiliated Hospital of Chongqing Medical University, Chongqing Key Laboratory of Ophthalmology, and Chongqing Eye Institute, Chongqing, P R China; 2 University Eye Clinic Maastricht, Maastricht, The Netherlands

## Abstract

This study aimed to investigate whether single nucleotide polymorphisms (SNPs) of five NLR family genes (NOD1, NOD2, NLRP1, NLRP3 and CIITA) are associated with Behcet’s disease (BD) in a Chinese Han population. The study was carried out in 950 BD patients and 1440 controls for 19 SNPs in the selected NLR genes. In the first-stage study, significantly decreased frequencies of the CIITA//rs12932187 C allele (Pc = 1.668E-02) and NOD1//rs2075818 G allele (Pc = 4.694E-02) were found in BD patients as compared to controls . After performing a second stage validation study and combination of data we confirmed the association of CIITA//rs12932187 and NOD1//rs2075818 with BD. In CIITA//rs12932187, the frequencies of the CC genotype and C allele were significantly lower in BD than in controls (Pc = 3.331E-06; Pc = 6.004E-07, respectively). In NOD1//rs2075818, the GG genotype and G allele showed significantly decreased frequencies in BD patients when compared to controls (Pc = 1.022E-02; Pc = 6.811E-05, respectively). Functional experiments showed that carriers with the CC genotype in CIITA//rs12932187 had a lower CIITA mRNA expression level and an enhanced IL-10 secretion as compared to GG and CG carriers. This study provides evidence that the CIITA and NOD1 gene are involved in the susceptibility to Behcet’s disease.

Behçet’s disease (BD) is a multifactorial disease which presents with oral aphthae, genital ulcerations, ocular inflammation, skin lesions and a pathognomonic pathergy test^1^. The etiology of BD is largely unknown, but cumulative evidence suggests that an excessive T-cell mediated inflammatory response is associated with disease activity^2^. Previous studies revealed that a number of genetic factors are involved in disease susceptibility, such as STAT3, STAT4, TLR2, miR-182, FAS and CD40 genes^3^,^4^,^5^,^6^,^7^,^8^,^9^.

BD is associated with important morbidity of which the intraocular inflammation may lead to serious visual handicap. The disease is currently being treated with corticosteroids and a variety of immunosuppressive agents. Further knowledge of the inflammatory pathways involved in the disease process may lead to the development of new drugs to target these disorders. One of the approaches currently used includes the analysis of the association of these diseases with gene polymorphisms of proteins involved in the immune or inflammatory response. Since BD may be triggered by an infectious process we focused on gene polymorphisms associated with the microbial immune response^8^.

Nucleotide-binding domain and leucine-rich repeat containing (NLRs) including at least 22 known proteins, exist in the cytosol and play an important role in the recognition of microbial products^10^. They are characterized by three structural domains: a NACHT- domain for oligomerization and activation of the NLRs, an LRR domain at the C-terminus which is responsible for recognition of microbial patterns, and a protein–protein interaction domain at the N-terminus that could be formed of a pyrin (PYD)-, caspase (CARD) or a baculo-virus inhibitor of apoptosis repeat (BIR) domain, triggering the signal transduction cascade^10^.

Few NLRs have been well characterized thus far, however, more recent studies demonstrate that variation in NLRs genes are associated with autoimmune or inflammatory disease. NOD2 was the first identified CD (Crohn’s disease) susceptibility gene^11^ and variations of NOD1 have been shown to confer risk to inflammatory bowel diseases (IBD) and CD^12^,^13^. NLRP3 variations have also been found to be associated with several autoimmune diseases including neonatal-onset multi-system inflammatory disease (NOMID), Muckle-Wells syndrome (MWS) and familial cold urticaria (FCU)^14^,^15^,^16^. NLRP1 has been found to confer risk to autoimmune rheumatoid arthritis (RA), Addison’s disease, type I diabetes and vilitigo^17^,^18^,^19^. Variation in CIITA was also found to be related to a number of autoimmune diseases such as RA, myocardial infarction and multiple sclerosis (MS)^20^,^21^.

On the basis of these previous studies, we conducted this research to investigate whether polymorphisms of NLR family genes including NOD1, NOD2, NLRP1, NLRP3 and CIITA gene were associated with BD.

## Results

### Clinical Features

Nineteen SNPs in five selected NLR genes (NOD1, NOD2, NLRP1, NLRP3 and CIITA) were genotyped successfully and all SNPs did not deviate from the Hardy-Weinberg equilibrium in controls. The case group comprised 186 women and 764 men, and the average age of the BD patients was 33.0031 ± 8.4 years. The healthy control cohort consisted of 1440 subjects (321 women, 1119 men), in which the average age was 35.9 ± 11.2 years. There were no statistical differences in age and sex between cases and controls (P > 0.05). The demographics and clinical features of BD patients enrolled in the study are summarized in Table 1.

### Genotype Results

In first stage study, the frequency of the CIITA//rs12932187 C allele (Pc = 1.668 × 10^−2^, OR = 0.713, 95% CI = 0.591–0.861) and NOD1//rs2075818 G allele (Pc = 4.694E-02, OR = 0.698, 95% CI = 0.562–0.868) were decreased in BD patients compared to controls (Table 2). The other seventeen SNPs did not show a significant association with BD (Supplementary Table S1). In the second stage, we tested another set of 566 BD patients and 870 healthy controls to confirm the result of the first stage study. After combination of the data, the frequencies of CC genotype and C allele in CIITA//rs12932187, were significantly decreased in the BD patients (Pc = 3.331 × 10^−6^, OR = 0.617, 95% CI = 0.519–0.735; Pc = 6.004 × 10^−7^, OR = 0.709, 95% CI = 0.629–0.799, respectively). In NOD1//rs2075818, the frequencies of the GG genotype and G allele were also decreased in the BD patients (Pc = 1.022E-02, OR = 0.536, 95% CI = 0.386–0.745; Pc = 6.811 × 10^−5^, OR = 0.720, 95% CI = 0.629–0.824, respectively).

### mRNA level and downstream inflammatory factors

**Figure 1:**
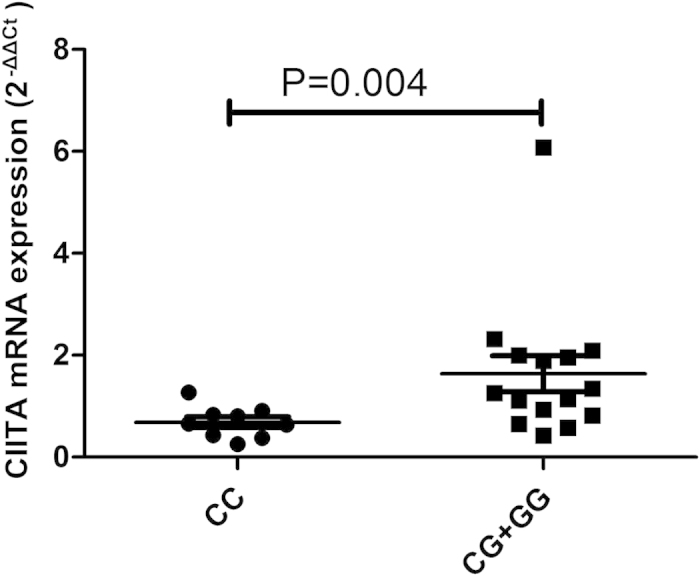
The influence of various rs12932187 genotypes on CIITA mRNA expression after stimulation with LPS. The expression of CIITA in PBMCs treated with LPS. PBMCs were obtained from healthy individuals with diverse genotypes of CIITA//rs12932187.

Because of the significant association of CIITA//rs12932187 and NOD1//rs2075818 with BD, we tested the expression of NOD1 and CIITA in PBMCs obtained from healthy individuals with known genotypes of the two SNPs. Real-time PCR did not show a detectable association between the various genotypes and the expression of NOD1 and CIITA when testing unstimulated PBMCs (Supplementary Fig. S1 and Supplementary Fig. S3). Following stimulation by LPS, carriers with the CC genotype in CIITA//rs12932187 had a lower mRNA expression of CIITA compared with the GG or CG genotype carriers (P = 0.004, Fig. 1). anti-CD3/anti-CD28 stimulation did not affect CIITA expression (Supplementary Fig. S2) and no effect on NOD1 mRNA expression was observed for the various rs2075818 genotypes by either normal or stimulated PBMCs (Supplementary Fig. S4 and Supplementary Fig. S5).

**Figure 2:**
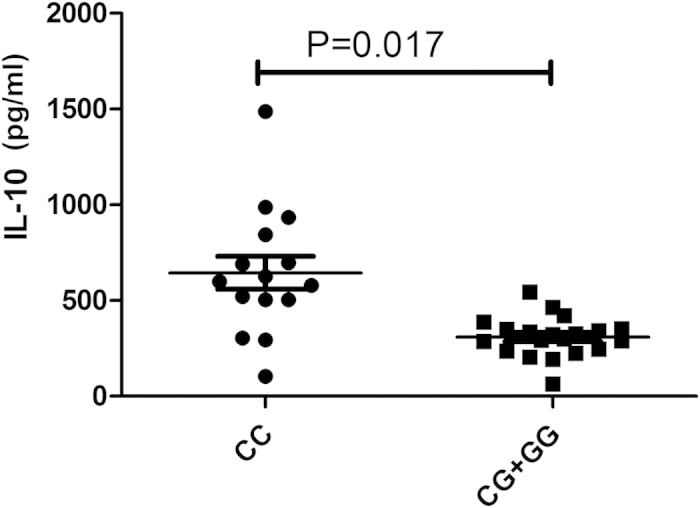
The influence of various rs12932187 genotypes on secretion of IL-10 after stimulation with LPS. The production of IL-10 in PBMCs obtained from healthy genotype controls. PBMCs were treated with LPS.

Since the different genotypes of CIITA//rs12932187 had an effect on CIITA mRNA expression, we decided to investigate whether the different genotypes influenced the cytokine response of PBMCs following LPS stimulation. We measured the PBMC expression levels of IL-6, IL-8, IL-10, IL-1β, TNF-α and MCP-1 by ELISA. These cytokines have all been shown to play a role in the development of BD as shown by earlier studies^22^,^23^. Carriers of the CC genotype had a higher secretion level of IL-10 as compared to GG and CG carriers (P = 0.017, Fig. 2). No significant effect on secretion levels of IL-6, IL-8, IL-1β, TNF-α and MCP-1 was found (Supplementary Fig. S6–S10).

## Discussion

In the present study, we investigated the associations of 19 SNPs in NOD1, NOD2, NLRP1, NLRP3 and CIITA with BD in a Chinese Han population. Two SNPs, rs12932187 of CIITA and rs2075818 of NOD1 contributed to the genetic susceptibility of BD. Functional studies showed that carriers of the CC genotype of CIITA//rs12932187 had a lower CIITA mRNA expression level and an increased IL-10 secretion by PBMCs as compared to GG and CG carriers.

CIITA acts as a transcriptional coactivator and has been associated with various inflammatory and autoimmune diseases^24^,^25^. CIITA mediates activated immune responses and its deficiency has been shown to cause Type II Bare lymphocyte syndrome (BLS)^26^. Variability in the CIITA gene has also been reported to be associated with several autoimmune and inflammatory diseases such as myocardial infarction (MI), rheumatoid arthritis (RA), type I diabetes (T1D) and multiple sclerosis (MS)^23^,^24^. A case-control study was performed in 1320 MS cases and 1363 independent healthy controls and the results showed that CIITA//rs4774 was associated with MS, particularly in DRB1*1501(+) cases^27^. Another study showed that the two SNPs rs4774 and rs6498122 of CIITA were associated with oral lichen planus (OLP)^28^. CIITA//rs8048002 was found to be associated with RA in a Swedish cohort^29^. CIITA//rs12932187 and rs11074938 were found to be susceptibility markers of nasal passages inflammation in asthma patients in a Japanese population^30^. In our study, only the CIITA//rs12932187 G allele and GG genotype were associated with BD risk. CIITA has been shown to function not only as a transcriptional regulator of MHC genes, but is also a transcriptional regulator of over 60 immunologically important genes, including IL-4, IL-10 and a number of thyroid-specific genes^24^,^25^. A study in CIITA-deficient (CIITA(−/−)) mice showed that CIITA negatively regulates the expression of IL-10 by DCs, which supports the findings in humans as presented here^31^. IL-10 is considered an immune regulatory cytokine which controls innate and adaptive immune responses^32^. Low IL-10 serum levels have been reported in Asian patients with BD^33^. The functional tests we performed showed that carriers with the CC genotype of CIITA//rs12932187 had a lower CIITA mRNA expression level and an enhanced IL-10 secretion as compared to GG and CG carriers. The protective effect of the C allele and CC genotype concerning BD development could thus be explained by the fact that these individuals produce more anti-inflammatory IL-10 in response to a microbial stimulus than carriers of the G allele. Further studies are needed to support this hypothesis.

NOD1 has been characterized as a critical regulator of innate immunity. Various studies have reported the association between NOD1 gene variants and autoimmune disease^10^. The NOD1//rs2075818 G allele was found to decrease the risk of CD and rs2907748 AA and AG genotypes showed a decreased frequency in UC^13^. These findings are in agreement with our study and could be due to the fact that BD as well as these inflammatory bowel disease are considered as an autoinflammatory disease caused by an aberrant response to a microbial agent. We were not able to detect a functional explanation for the association with NOD1//rs2075818.

NLRP1 and NLRP3 have been shown to play an important role in the processing of pivotal pro-inflammatory cytokines such as IL-1β and IL-18^14^,^16^. Gene variants of NLRP3 have been shown to be associated with Psoriatic Juvenile Idiopathic Arthritis in a Caucasian population^34^. Moreover, genetic variants of NLRP1 were observed to confer risk for the development of vitiligo^35^. Nevertheless, our study did not find an association between NLRP1 and NLRP3 and BD in a Chinese Han population. NOD2, that was already identified as a CD-susceptibility gene^11^, was not associated with BD.

Our study has some limitations. Since the subjects in our study were all Chinese Han, the conclusions of the study are only valid to the Chinese Han population and should be studied and replicated in other ethnic groups. Furthermore, all the BD patients in this study were recruited from ophthalmology departments and a selection bias in our patient population may be present. Whether our findings can be generalized to other uveitis entities is not known and deserves further study. We did test the SNPs described in this study on uveitis patients with Vogt Koyanagi Harada syndrome but did not observe statistically significant associations (data not shown).

In conclusion, this study for the first time reports an association of CIITA//rs12932187 and NOD1//rs2075818 with susceptibility to BD in a Chinese Han population. A functional variant of CIITA//12932187 was shown to regulate CIITA expression and IL-10 production.

## Materials and Methods

### Study population

In the first stage of this study, a total of 384 BD patients and 576 controls were enrolled to identify disease susceptibility loci in the family of NLR genes. In the second (confirmatory) stage, another set of 566 BD patients and 864 controls were added to replicate the susceptible SNPs found in the first stage study.

All blood samples were enrolled at the Zhongshan Ophthalmic Center (Guangzhou, China) and the First Affiliated Hospital of Chongqing Medical University (Chongqing, China) from November 2006 to February 2015. The diagnosis of BD patients is based on the criteria of the International Study Group for BD^36^. This study obtained the approval of the Local Ethics Research Council. before the collection of blood, all the investigated individuals had signed the informed consent.

### Ethical considerations

Before the collection of blood, all the investigated individuals had signed the informed consent. The investigation protocols obtained the approval of the Clinical Research Ethics Committee of the Zhongshan Ophthalmic Center of Sun Yat-sen University and the First Affiliated Hospital of Chongqing Medical University. All experiments were conducted in accordance with the approved guidelines and regulations. This study was conducted according to the tenets of the Declaration of Helsinki.

### SNP selection

We selected nineteen SNPs of NLR family genes including NOD1//(rs2075818, rs2907748, rs2907749), NOD2//(rs8057431, rs3135499), NLRP1//(rs6502867, rs878329, rs12150220, rs8079034), NLRP3//(rs10754558, rs10925019, rs4925648, rs3806265, rs2027432) and CIITA//(rs12932187, rs1107438, rs8048002, rs6498122, rs4774) on the basis of three standards : 1. According to previous reports, the relevant SNP had been proved to be associated with an autoimmune or auto-inflammatory disease. 2. Allele or genotype data existed in the National Center of Biotechnology Information (NCBI) single nucleotide polymorphisms (dbSNP) database in the Chinese Han population. 3. All SNPs had to have a minor allele frequency (MAF) that was larger than 0.05 and an R^2^ threshold of 0.80; The R^2^ value reflects the degree to which captured alleles are in linkage disequilibrium (LD) with the tag SNP. Genotyping data for each of the 5 NLRP genes (Chinese Han population) was downloaded from the HapMap Web site (http://hapmap.ncbi.nlm.nih.gov/index.html.en) .

### DNA extraction and genotyping

Genomic DNA was extracted from the blood of patients and healthy individuals using the QIAmp DNA Blood Mini Kit (Qiagen Inc., Valencia, CA, USA), all the samples were stored at −80 °C until used. All SNPs except NLRP3//rs10925019 were genotyped by polymerase chain reaction-restriction fragment length polymorphism (PCR-RFLP). All the primers were designed using primer software 5.0 (Premier Biosoft International, Palo Alto, CA, USA). Primers and restriction enzymes are shown in Table 3. NLRP3//rs10925019 (TaqMan assay ID: C_26646027_10) genotyping was performed on the Applied Biosystems 7500 Real-Time PCR system using TaqMan^®^ SNP assay (Applied Biosystems, CA, USA). The analysis was performed by TaqMan Genotyper Software. To verify the accuracy of genotyping, direct sequencing was carried out (Beijing Biomed Co. Ltd. China) using randomly selected samples (10% of all samples). The genotyping success rate was above 95%.

### Real-time PCR

In this study, peripheral blood mononuclear cells (PBMCs) were obtained from healthy controls by Ficoll-Hypaque density-gradient centrifugation. Cells were stimulated with or without anti-CD3 (0.5ug/ml) and anti-CD28 antibodies (0.1ug/ml, eBioscience, San Diego, CA, USA) to analog antigen presentation or lipopolysaccharide (LPS, 5ug/ml, Fluka, Buchs, Switzerland) to analog an inflammatory signal for 72 hours at a density of 1 × 10^6^ cells/ml. RNA was acquired from the cultured cells by TRIzol (Invitrogen), after reserve transcription (transcription kit, Takara Biotechnology Co. Ltd., Dalian, China.), mRNA expression of NOD1 gene (forward: 5′ TTGACCACCCTGAGTCTTGC 3′, reserve: 5′ TCATTTTGGGTCAGCCACAG 3′) and CIITA gene (forward: 5′ TGAGGCTGTGTGCTTCTGAG 3′, reserve: 5′ ACACTGTGAGCTGCCTTGG 3′) was measured by using real-time PCR equipment with a commercial dye kit (Applied Biosystems), β-Actin was selected as the internal reference gene and its expression was detected by the following primers: forward 5′-GGATGCAGAAGGAGATCACTG -3′ and reverse 5′-CGATCCACACGGAGTACTT-3′. Data were normalized to mRNA beta-actin and expression levels were calculated by the 2^−△△^ method.

### Cytokine Measurements

The human Duoset enzyme-linked immunosorbent assay (ELISA) development kit (R&D System, Minneapolis, MN) was used to measure the concentration of IL-6, IL-8, IL-10, IL-1β, TNF-α and MCP-1 in cell culture supernatant in accordance with the manufacturers’ instructions.

### Statistical analysis

Differences in alleles and genotypes of all SNP variations were evaluated by the Fisher’s exact test or X^2^ test using SPSS (version 17.0; SPSS Inc, Chicago, IL). The p values were corrected with the Bonferroni correction method and a Pc <0.05 was considered to be significant. The X^2^ test was used to determine the Hardy-Weinberg equilibrium (HWE). The independent samples t test or nonparametric Mann-Whitney U test was used to compare CIITA, NOD1 and cytokine (IL-6, IL-8, IL-10, TNF-α, IL-1βand MCP-1) expression levels among three genotype groups.

## Additional Information

**How to cite this article**: Li, L. *et al*. Genetic Variations of NLR family genes in Behcet's Disease. *Sci. Rep*. **6**, 20098; doi: 10.1038/srep20098 (2016).

## Supplementary Material

Supplementary Information

## Figures and Tables

**Table 1:** Clinical Features, Age and Gender Distribution in Controls as well as Patients with Ocular BD.

Clinical Features	Total	%
Ocular BD Patients	950	
Age at onset, year ± SD	33.1 ± 8.4	
Female	950	19.6
Male	755	80.4
Uveitis	950	100
Oral ulcer	950	100
Genital ulcer	466	49.1
Skin lesions	570	60.0
Pathergy reaction	231	24.3
Hypopyon	202	21.3
Arthritis	151	15.9
Controls	1440	
Age at onset, year ± SD	35.9 ± 11.2	
Female	321	22.3
Male	1119	77.7

SD = standard deviation.

BD = Behcet disease.

**Table 2:** Polymorphisms of *NOD1//rs2075818* and *CIITA//rs12932187* in Behcet’s Disease.

SNPs	Stage	Genotype	Cases, No.		Controls, No.		*P* Value	*P*c Value	OR (95% CI)
		Allele	(Frequency)		(Frequency)				
rs12932187 (*CIITA*)	First^*a*^	CC	113	(0.298)	233	(0.405)	8.212E-04	4.927 E-02	0.625 (0.474 to 0.824)
		CG	204	(0.538)	280	(0.486)	0.115	NS	1.232 (0.950 to 1.598)
		GG	62	(0.164)	63	(0.109)	0.015	NS	1.593 (1.093 to 2.323)
		C	430	(0.567)	746	(0.648)	4.169E-04	1.668E-02	0.713 (0.591 to 0.861)
		G	328	(0.433)	406	(0.352)	4.169E-04	1.668E-02	1.402 (1.162 to 1.691)
	Replication^*b*^	CC	170	(0.304)	359	(0.416)	1.878E-05	1.120E-02	0.612 (0.489 to 0.767)
		CG	298	(0.533)	408	(0.473)	0.028	NS	1.270 (1.026 to 1.573)
		GG	91	(0.163)	95	(0.110)	0.004	NS	1.570 (1.152 to 2.140)
		C	638	(0.571)	1126	(0.653)	9.586E-06	3.835E-04	0.706 (0.605 to 0.824)
		G	480	(0.429)	598	(0.347)	9.586E-06	3.835E-04	1.417 (1.214 to 1.653)
	Combined^*c*^	CC	283	(0.302)	592	(0412)	5.552E-08	3.331E-06	0.617 (0.519 to 0.735)
		CG	502	(0.535)	688	(0.478)	0.007	NS	1.255 (1.064 to 1.480)
		GG	153	(0.163)	158	(0.110)	1.693E-04	1.016E-05	1.579 (1.243 to 2.006)
		C	1068	(0.569)	1872	(0.651)	1.501E-08	6.004E-07	0.709 (0.629 to 0.799)
		G	808	(0.431)	1004	(0.349)	1.501E-08	6.004E-07	1.411 (1.252 to 1.589)
rs2075818 (*NOD1*)	First	CC	235	(0.618)	299	(0.525)	0.004	NS	1.469 (1.128 to 1.913)
		CG	130	(0.342)	217	(0.381)	0.226	NS	0.846 (0.645 to 1.109)
		GG	15	(0.040)	54	(0.094)	0.001	NS	0.393 (0.218 to 0.707)
		C	600	(0.789)	825	(0.724)	1.173E-03	4.694E-02	1.432 (1.152 to 1.780)
		G	160	(0.211)	315	(0.276)	1.173E-03	4.694E-02	0.698 (0.562 to 0.868)
	Replication^*b*^	CC	328	(0.588)	431	(0.502)	0.002	NS	1.416 (1.142 to 1.756)
		CG	193	(0.346)	341	(0.397)	0.052	NS	0.803 (0.644 to 1.002)
		GG	37	(0.066)	87	(0.101)	0.023	NS	0.630 (0.422 to 0.940)
		C	849	(0.761)	1203	(0.700)	4.29E-04	1.715E-02	1.361 (1.146 to 1.617)
		G	267	(0.239)	515	(0.300)	4.29E-04	1.715E-02	0.735 (0.619 to 0.872)
	Combined^*c*^	CC	563	(0.601)	730	(0.511)	1.941E-05	1.164E-03	1.438 (1.217 to 1.699)
		CG	323	(0.344)	558	(0.390)	0.023	NS	0.820 (0.691 to 0.973)
		GG	52	(0.055)	141	(0.099)	1.703E-04	1.022E-02	0.536 (0.386 to 0.745)
		C	1449	(0.772)	2028	(0.710)	1.703E-06	6.811E-05	1.389 (1.214 to 1.589)
		G	427	(0.228)	830	(0.290)	1.703E-06	6.811E-05	0.720 (0.629 to 0.824)

NS = no significant difference; OR = odds ratio; *P*_c_ = *P* value with Bonferroni correction.

SNP = single nucleotide polymorphism.

^a^First stage (stage 1), case-to-control ratio: 380:576; ^b^Replication stage (stage 2), case-to-control ratio: 559:862; ^c^Combined stage(a+b), case-to-control ratio: 939:1438.

**Table 3:** Primers and restriction enzymes used for RFLP analysis of the NOD1, NOD2, NLRP1, NLRP3 and CIITA genes.

Gene	SNP number	Primers	Restriction enzyme
NOD1	rs2075818	GCAATCGGGAACTTCTGGTCACT	HaeIII
		GGGGCAGGCACACACAATCTC	
	rs2907748	AAGGCTCTCCAGCTATGCAGAT	PvuIII
		GTGGGCTCCTCTACAGGCA	
	rs2907749	CCCCCACACACACAGCAGGTT	BccI
		GCTGGAGGCTGACTGTGTGTGAC	
NOD2	rs8057431	CTGACTGAGGCAGCGGGAGTTTA	DraI
		CAGGAGACCAAGGCAGGAAGACC	
	rs3135499	CGGCCTCTCACAAAAGACCGGAT	BamHI
		GGAATGGCCTGGATGGATGAGT	
NLRP1	rs8079034	CGCAGACAAAGGTCCTTAGGTA	BstEII
		AACTTGAAGGGAAGTCTAGCAGT	
	rs12150220	GCTTGGAGACTCATGGTCTG	BseRI
		CCCTCTACTTCAACATGGTTTTCA	
	rs6502867	GGACAGAATTAAGACTGATAA	ApaLI
		TTATCAGTCTTAATTCTGTCC	
	rs878329	CCGGGCTGCATCAACCTTCT	Bsp1286I
		GCCCCAACCACCAACATGAGAC	
NLRP3	rs10754558	CAGGACAATGACAGCATCGGGTGTTGAT	MboI
		GCTGCCATAAAATTTCAACATAA	
	rs4925648	TTCCTGGTTCTAAACCCCTCTG	PstI
		CACAGGCTAGGCACTCACT	
	rs3806265	TTGGCAGGTGGACAGCAGCA	PvuII
		GACCCCAAACATCCCCCAAATCA	
	rs2027432	CACCATACACCTTTTTTCTCGGGC	BstEII
		GGGCCTCCATTTTCTCATCTGTG	
CIITA	rs12932187	TGCCCCTGAAGAAGTCGTTT	TaqI
		CTTAAGGCTGCACCCAACCAC	
	rs1107438	ACAGTCATCATCTTCCCCATTTTAC	HinfI
		GCCCTCAGTTATTGTTTTCAGAGAT	
	rs8048002	CACCATACACCTTTTTTCTCGGGC	HaeIII
		GGGCCTCCATTTTCTCATCTGTG	
	rs6498122	GTCCCTCAGTTTTGCTCCTATC	Csp6I
		GTCCTCTCCCTCAATAATATGGT	
	rs4774	TCCCCTGCCATTGCTTGA	Hin1I
		AACCTCGGAGCAGCTTCTTCT	
