# Corrigendum: Genetic Variations of NLR family genes in Behcet’s Disease

**DOI:** 10.1038/srep26423

**Published:** 2016-05-31

**Authors:** Lin Li, Hongsong Yu, Yanni Jiang, Bolin Deng, Lin Bai, Aize Kijlstra, Peizeng Yang

Scientific Reports 6: Article number: 2009810.1038/srep20098; published online: 02012016; updated: 05312016

This Article contains errors.

In Table 1, the number of Female ‘186’ and Male ‘764’ patients enrolled in this study is incorrectly given as ‘950’ and ‘755’ respectively.

In the Results section,

“The case group comprised 186 women and 764 men, and the average age of the BD patients was 33.0031 ± 8.4 years.”

should read:

“The case group comprised 186 women and 764 men, and the average age of the BD patients was 33.1 ± 8.4 years.”

In the Results section under subheading ‘Genotype Results’,

“In first stage study, the frequency of the CIITA//rs12932187 C allele (Pc = 1.668 × 10^−2^, OR = 0.713, 95% CI = 0.591–0.861) and NOD1//rs2075818 G allele (Pc = 4.694E-02, OR = 0.698, 95% CI = 0.562–0.868) were decreased in BD patients compared to controls (Table 2).”

should read:

“In first stage study, the frequency of the CIITA//rs12932187 C allele (Pc = 1.668 × 10^−2^, OR = 0.713, 95% CI = 0.591–0.861) and NOD1//rs2075818 G allele (Pc = 4.694 × 10^−2^, OR = 0.698, 95% CI = 0.562–0.868) were decreased in BD patients compared to controls (Table 2).”

“In NOD1//rs2075818, the frequencies of the GG genotype and G allele were also decreased in the BD patients (Pc = 1.022E-02, OR = 0.536, 95% CI = 0.386–0.745; Pc = 6.811 × 10^−5^, OR = 0.720, 95% CI = 0.629–0.824, respectively).”

should read:

“In NOD1//rs2075818, the frequencies of the GG genotype and G allele were also decreased in the BD patients (Pc = 1.022 × 10^−2^, OR = 0.536, 95% CI = 0.386–0.745; Pc = 6.811 × 10^−5^, OR = 0.720, 95% CI = 0.629–0.824, respectively).”

In Table 3, the Restriction enzyme ‘PvuII’ for gene NOD1 ‘rs2907748’ is incorrectly given as ‘PvuIII’.

