# Supplementary material for: Genetic Variations of NLR family genes in Behcet’s Disease
Source: Sci Rep. 2016 Feb 1;6:20098. doi: 10.1038/srep20098 (PMC4735577; doi:10.1038/srep20098)
Supplement: Supplementary Information [file srep20098-s1.doc]

**Genetic Variations of NLR family genes in Behcet's Disease**

# Lin Li^1^, Hongsong Yu^1^, Yanni Jiang^1^, Bolin Deng^1^, Lin Bai^1^, Aize Kijlstra^2^, and Peizeng Yang^1^

# **^1^The First Affiliated Hospital of Chongqing Medical University, Chongqing Key Laboratory of Ophthalmology, and Chongqing Eye Institute, Chongqing, P R China. ^2^**University Eye Clinic Maastricht, Maastricht, The Netherlands.

Corresponding author: Prof. Dr. Peizeng Yang, MD, PhD

The First Affiliated Hospital of Chongqing Medical University, Youyi Road 1, Chongqing, 400016, P. R. China

Fax & Phone: 0086-23-89012851

E-mail address: [peizengycmu@126.com](mailto:peizengycmu@126.com)

**Supplementary Information**

Supplementary Table S1. Polymorphisms of *NOD1*, *NOD2*, *NLRP1*, *NLRP3* and *CIITA* Genes in Behcet's Disease

|  |  | Genotype | Cases, No. | | Controls, No. | |  |  |  |  |
| --- | --- | --- | --- | --- | --- | --- | --- | --- | --- | --- |
| SNPs | Stage | Allele | (Frequency) | | (Frequency) | | *P* Value | *P*c Value | OR (95% CI) |  |
|  |  |  |  |  |  |  |  |  |  |  |
| rs6498122 (*CIITA*) | First | AA | 259 | (0.678) | 380 | (0.663) | 0.633 | NS | 1.069 (0812 to 1.409) |  |
|  |  | AG | 110 | (0.288) | 167 | (0.292) | 0.907 | NS | 0.983 (0.739 to 1.308) |  |
|  |  | GG | 13 | (0.034) | 26 | (0.045) | 0.386 | NS | 0.741 (0.376 to 1.461) |  |
|  |  | A | 628 | (0.822) | 927 | (0.809) | 0.471 | NS | 1.091 (0.861 to 1.382) |  |
|  |  | G | 136 | (0.178) | 219 | (0.191) | 0.471 | NS | 0.917 (0.723 to 1.162) |  |
| rs1107438 (*CIITA*) | First | AA | 88 | (0.232) | 147 | (0.256) | 0.390 | NS | 0.875 (0.646 to 1.185) |  |
|  |  | AG | 182 | (0.479) | 268 | (0.467) | 0.715 | NS | 1.050 (0.810 to 1.361) |  |
|  |  | GG | 110 | (0.289) | 159 | (0.277) | 0.675 | NS | 1.063 (0.798 to 1417) |  |
|  |  | A | 358 | (0.471) | 562 | (0.490) | 0.429 | NS | 0.929 (0.773 to 1116) |  |
|  |  | G | 402 | (0.529) | 586 | (0.510) | 0.429 | NS | 1.077 (0.896 to 1.294) |  |
| rs8048002 (*CIITA*) | First | CC | 34 | (0.089) | 36 | (0.065) | 0.152 | NS | 1.426 (0.876 to 2.323) |  |
|  |  | CT | 143 | (0.375) | 232 | (0.414) | 0.231 | NS | 0.849 (0.650 to 1.109) |  |
|  |  | TT | 204 | (0.536) | 292 | (0.521) | 0.673 | NS | 1.058 (0.815 to 1.373) |  |
|  |  | C | 211 | (0.277) | 304 | (0.271) | 0.794 | NS | 1.028 (0.836 to 1.263) |  |
|  |  | T | 551 | (0.723) | 816 | (0.729) | 0.794 | NS | 0.973 (0.792 to 1.196) |  |
| rs4774 (*CIITA*) | First | CC | 3 | (0.009) | 3 | (0.005) | 0.618 | NS | 1.500 (0.301 to 7.471) |  |
|  |  | CG | 53 | (0.139) | 89 | (0.156) | 0.470 | NS | 0.873 (0.604 to 1.262) |  |
|  |  | GG | 325 | (0.853) | 478 | (0.839) | 0.548 | NS | 1.117 (0.779 to 1.602) |  |
|  |  | C | 59 | (0.077) | 95 | (0.083) | 0.644 | NS | 0.923 (0.658 to 1.295) |  |
|  |  | G | 703 | (0.923) | 1045 | (0.917) | 0.644 | NS | 1.083 (0.772 to 1.520) |  |
| rs2907749 (*NOD1*) | First | AA | 193 | (0.504) | 275 | (0.489) | 0.660 | NS | 1.060 (0.631 to 1.075) |  |
|  |  | AG | 171 | (0.446) | 247 | (0.440) | 0.832 | NS | 1.029 (0.827 to 1.373) |  |
|  |  | GG | 19 | (0.050) | 40 | (0.071) | 0.179 | NS | 0.681 (0.874 to 1.708) |  |
|  |  | A | 557 | (0.727) | 797 | (0.709) | 0.392 | NS | 1.093 (0.718 to 1.032) |  |
|  |  | G | 209 | (0.273) | 327 | (0.291) | 0.392 | NS | 0.915 (0.969 to 1.393) |  |
| rs2907748 (*NOD1*) | First | CC | 205 | (0.541) | 311 | (0.540) | 0.977 | NS | 1.004 (0.774 to 1.302) |  |
|  |  | CT | 157 | (0.414) | 233 | (0.405) | 0.765 | NS | 1.041 (0.800 to 1.355) |  |
|  |  | TT | 17 | (0.045) | 32 | (0.055) | 0.463 | NS | 0.798 (0.437 to 1.459) |  |
|  |  | C | 567 | (0.748) | 855 | (0.742) | 0.775 | NS | 1.031 (0.835 to 1.273) |  |
|  |  | T | 191 | (0.252) | 297 | (0.258) | 0.775 | NS | 0.970 (0.786 to 1.197) |  |
| rs3135499  *(NOD2)* | First | AA | 223 | (0.594) | 291 | (0.511) | 0.012 | NS | 1.402 (1.077 to 1.824) |  |
|  |  | AC | 136 | (0.363) | 240 | (0.422) | 0.069 | NS | 0.780 (0.596 to 1.020) |  |
|  |  | CC | 16 | (0.043) | 38 | (0.067) | 0.118 | NS | 0.623 (0.342 to 1.134) |  |
|  |  | A | 597 | (0.796) | 822 | (0.722) | 0.009 | NS | 1.332 (1.074 to 1.651) |  |
|  |  | C | 183 | (0.224) | 316 | (0.278) | 0.009 | NS | 0.751 (0.606 to 0.931) |  |
| rs8057431 *(NOD2)* | First | AA | 176 | (0.473) | 279 | (0.488) | 0.660 | NS | 0.943 (0.726 to 1.225) |  |
|  |  | AG | 158 | (0.425) | 253 | (0.442) | 0.595 | NS | 0.931 (0.715 to 1.212) |  |
|  |  | GG | 38 | (0.102) | 40 | (0.070) | 0.079 | NS | 1.513 (0.951 to 2.408) |  |
|  |  | A | 510 | (0.685) | 811 | (0.709) | 0.278 | NS | 0.895 (0.732 to 1.094) |  |
|  |  | G | 234 | (0.315) | 333 | (0.291) | 0.278 | NS | 1.117 (0.914 to 1.366) |  |
| rs6502867 *(NLRP1)* | First | TT | 335 | (0.877) | 515 | (0.904) | 0.194 | NS | 0.761 (0.504 to 1.150) |  |
|  |  | CT | 47 | (0.123) | 55 | (0.096) | 0.194 | NS | 1.314 (0.869 to 1.985) |  |
|  |  | T | 717 | (0.938) | 1085 | (0.952) | 0.207 | NS | 0.773 (0.518 to 1.154) |  |
|  |  | C | 47 | (0.062) | 55 | (0.048) | 0.207 | NS | 1.293 (0.866 to 1.930) |  |
| rs878329 (*NLRP1*) | First | CC | 6 | (0.016) | 15 | (0.027) | 0.252 | NS | 0.576 (0.221 to 1.497) |  |
|  |  | CG | 115 | (0.299) | 173 | (0.309) | 0.743 | NS | 0.954 (0.719 to 1.265) |  |
|  |  | GG | 263 | (0.685) | 371 | (0.664) | 0.495 | NS | 1.101 (0.834 to 1.454) |  |
|  |  | C | 127 | (0.165) | 203 | (0.182) | 0.363 | NS | 0.893 (0.700 to 1.139) |  |
|  |  | G | 641 | (0.835) | 915 | (0.818) | 0.363 | NS | 1.120 (0.878 to 1.429) |  |
| rs12150220 (*NLRP1*) | First | AA | 326 | (0.874) | 506 | (0.891) | 0.429 | NS | 0.850 (0.568 to 1.273) |  |
|  |  | AT | 47 | (0.126) | 62 | (0.109) | 0.429 | NS | 1.177 (0.786 to 1.762) |  |
|  |  | A | 699 | (0.937) | 1074 | (0.945) | 0.444 | NS | 0.859 (0.581 to 1.269) |  |
|  |  | T | 47 | (0.063) | 62 | (0.055) | 0.444 | NS | 1.165 (0.788 to 1.722) |  |
| rs8079034 *(NLPP1)* | First | CC | 223 | (0.596) | 357 | (0.621) | 0.447 | NS | 0.902 (0.691 to 1.177) |  |
|  |  | CT | 133 | (0.356) | 193 | (0.336) | 0.527 | NS | 1.092 (0.831 to 1.436) |  |
|  |  | TT | 18 | (0.048) | 25 | (0.043) | 0.736 | NS | 1.112 (0.598 to 2.069) |  |
|  |  | C | 579 | (0.774) | 907 | (0.789) | 0.450 | NS | 0.918 (0.735 to 1.146) |  |
|  |  | T | 169 | (0.226) | 243 | (0.211) | 0.450 | NS | 1.089 (0.872 to 1.361) |  |
| rs3806265 *(NLPP3)* | First | CC | 123 | (0.323) | 187 | (0.336) | 0.680 | NS | 0.943 (0.715 to 1.245) |  |
|  |  | CT | 169 | (0.444) | 258 | (0.463) | 0.553 | NS | 0.924 (0.711 to 1.200) |  |
|  |  | TT | 89 | (0.233) | 112 | (0.201) | 0.233 | NS | 1.211 (0.884 to 1.659) |  |
|  |  | C | 415 | (0.545) | 632 | (0.567) | 0.331 | NS | 0.912 (0.758 to 1.098) |  |
|  |  | T | 347 | (0.455) | 482 | (0.433) | 0.331 | NS | 1.096 (0.911 to 1.320) |  |
| rs4925648  *(NLRP3)* | First | CC | 224 | (0.583) | 316 | (0.551) | 0.316 | NS | 1.143 (0.880 to 1.484) |  |
|  |  | CT | 142 | (0.370) | 228 | (0.397) | 0.393 | NS | 0.890 (0.682 to 1.162) |  |
|  |  | TT | 18 | (0.047) | 30 | (0.052) | 0.708 | NS | 0.892 (0.490 to 1.624) |  |
|  |  | C | 590 | (0.768) | 860 | (0.749) | 0.340 | NS | 1.110 (0.896 to 1.375) |  |
|  |  | T | 178 | (0.232) | 288 | (0.251) | 0.340 | NS | 0.901 (0.727 to 1.116) |  |
| rs10754558 *(NLRP3)* | First | GG | 96 | (0.252) | 126 | (0.222) | 0.272 | NS | 1.186 (0.875 to 1.608) |  |
|  |  | CC | 180 | (0.474) | 278 | (0.489) | 0.634 | NS | 0.939 (0.724 to 1.218) |  |
|  |  | CC | 104 | (0.274) | 164 | (0.289) | 0.614 | NS | 0.928 (0.695 to 1.240) |  |
|  |  | G | 372 | (0.489) | 530 | (0.467) | 0.327 | NS | 1.096 (0.912 to 1.318) |  |
|  |  | C | 388 | (0.511) | 606 | (0.533) | 0.327 | NS | 0.912 (0.759 to 1.096) |  |
| rs10925019 (*NLRP3*) | First | CC | 212 | (0.563) | 305 | (0.536) | 0.385 | NS | 1.123 (0.864 to 1.460) |  |
|  |  | CT | 154 | (0.410) | 222 | (0.389) | 0.536 | NS | 1.087 (0.834 to 1.418) |  |
|  |  | TT | 10 | (0.027) | 43 | (0.075) | 0.001 | NS | 0.335 (0.166 to 0.675) |  |
|  |  | C | 578 | (0.769) | 832 | (0.730) | 0.058 | NS | 1.230(0.993 to 1.523) |  |
|  |  | T | 174 | (0.231) | 308 | (0.270) | 0.058 | NS | 0.813 (0.657 to 1.007) |  |
| rs2027432 *(NLRP3)* | First | CC | 343 | (0.893) | 500 | (0.874) | 0.370 | NS | 1.205 (0.802 to 1.810) |  |
|  |  | CT | 38 | (0.099) | 69 | (0.121) | 0.297 | NS | 0.801 (0.527 to 1.217) |  |
|  |  | TT | 3 | (0.008) | 3 | (0.005) | 0.622 | NS | 1.493 (0.300 to 7.438) |  |
|  |  | C | 724 | (0.943) | 1069 | (0.934) | 0.463 | NS | 1.154 (0.786 to 1.695) |  |
|  |  | T | 44 | (0.057) | 75 | (0.066) | 0.463 | NS | 0.866 (0.590 to 1.272) |  |

NS = no significant difference; OR = odds ratio; *P*_c_ = *P* value with Bonferronicorrection; SNP = single nucleotide polymorphism;

**
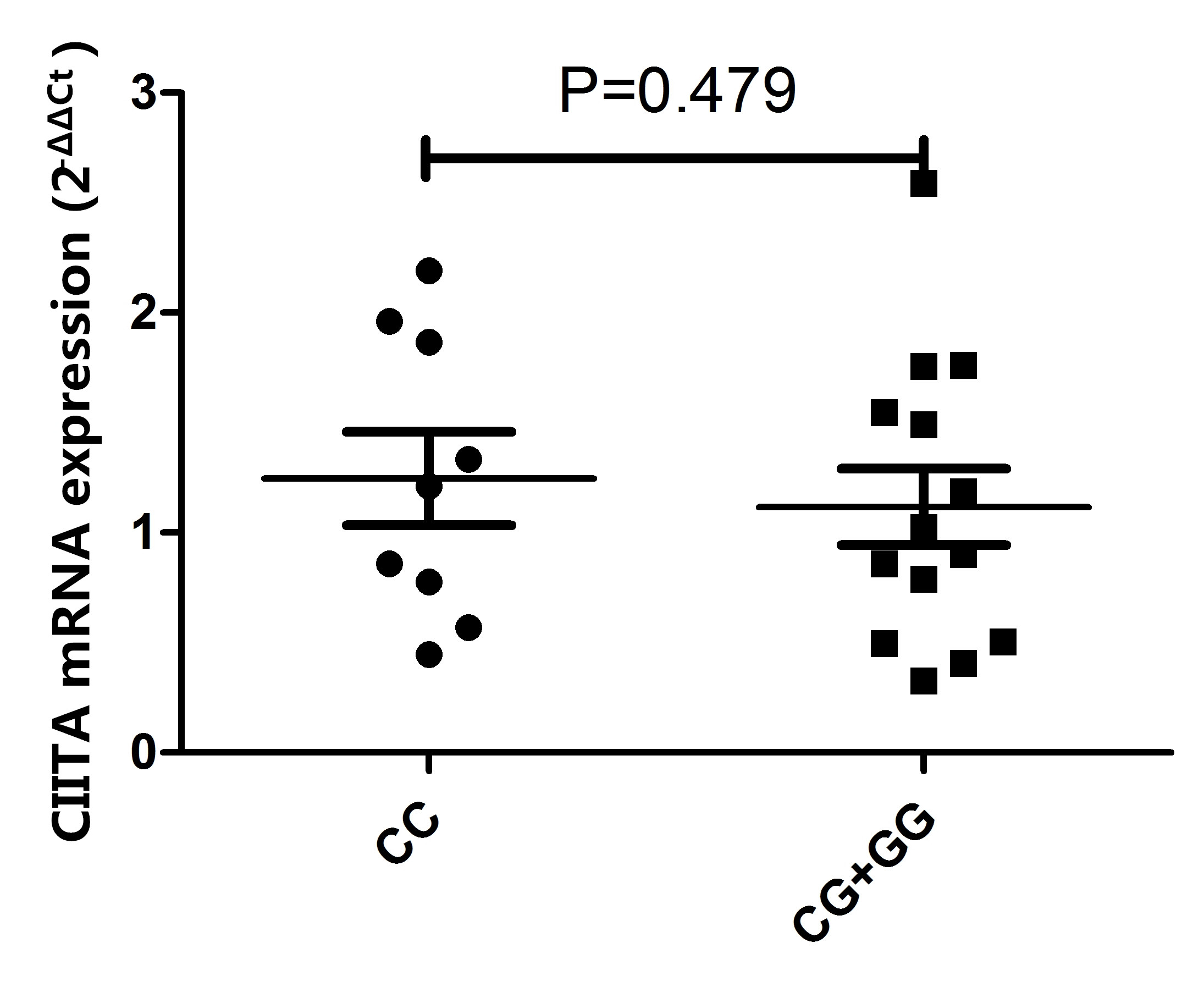
**

**Supplementary Figure S1.** The influence of various rs12932187 genotypes on

CIITA mRNA expression by unstimulated PBMCs.

**
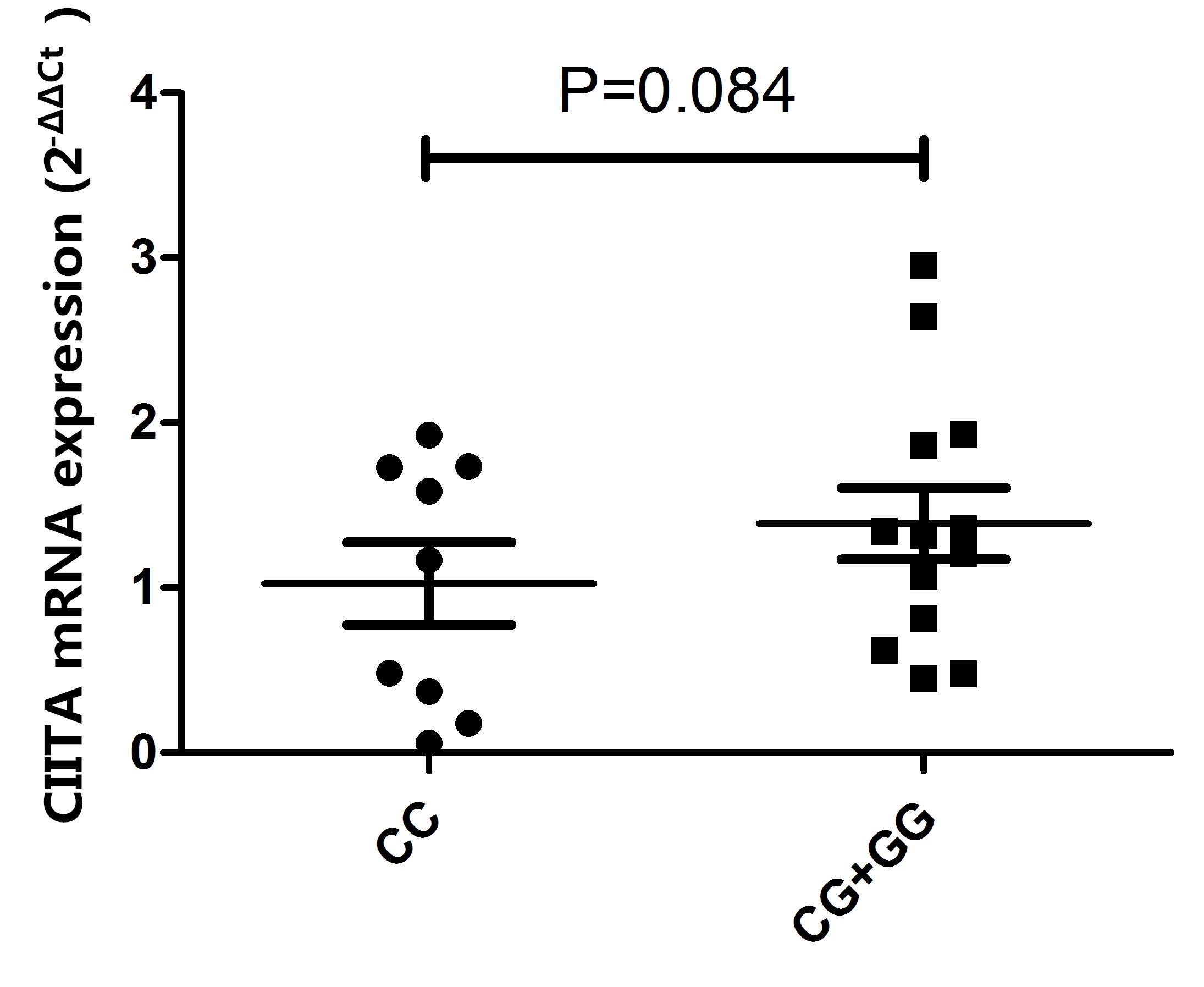
**

**Supplementary Figure S2.** The influence of various rs12932187 genotypes on

CIITA mRNA expression after stimulation with anti-CD3/CD28

antibodies.

**
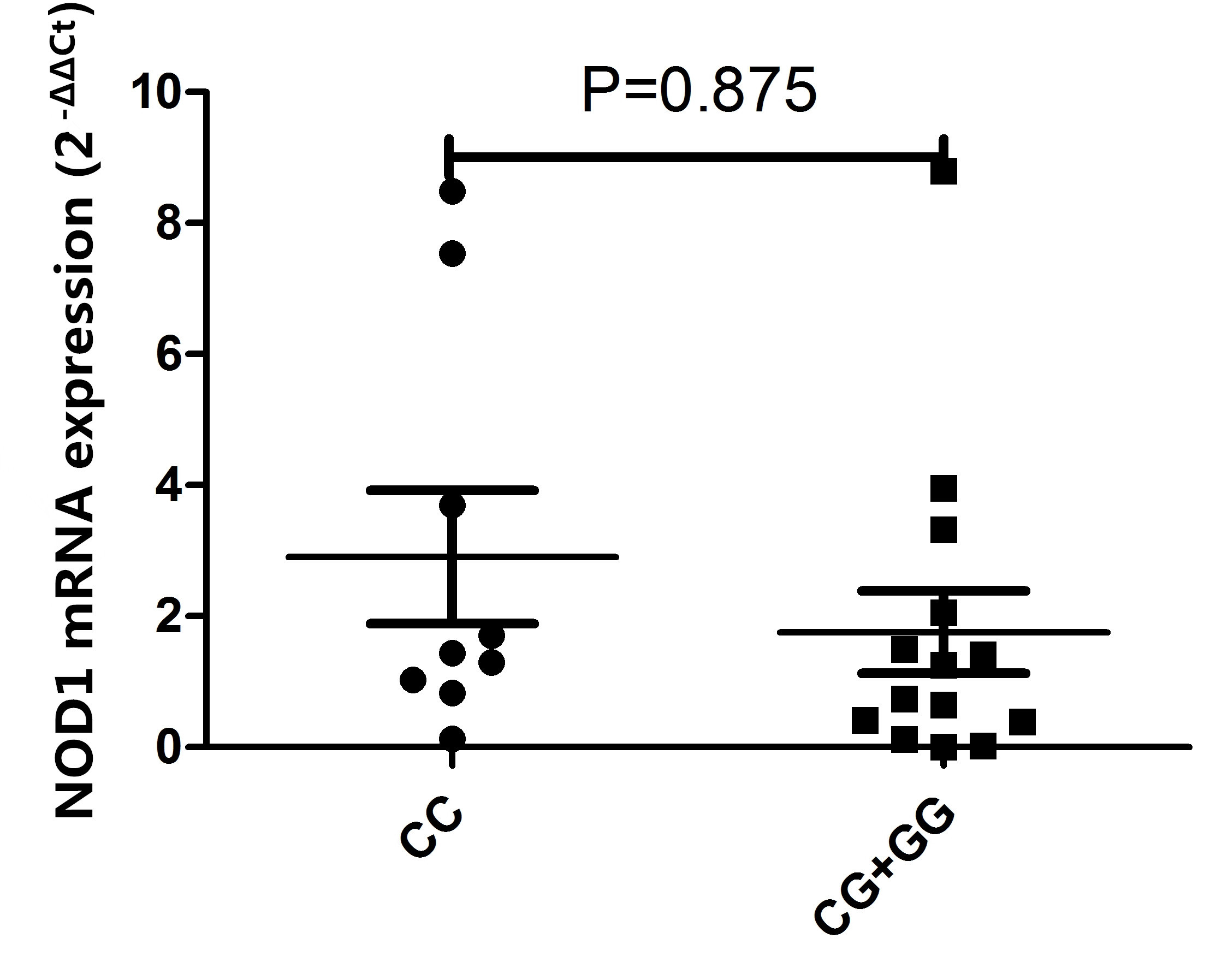
**

**Supplementary Figure S3.** The influence of various rs2075818 genotypes on

NOD1 mRNA expression by unstimulated PBMCs.

**
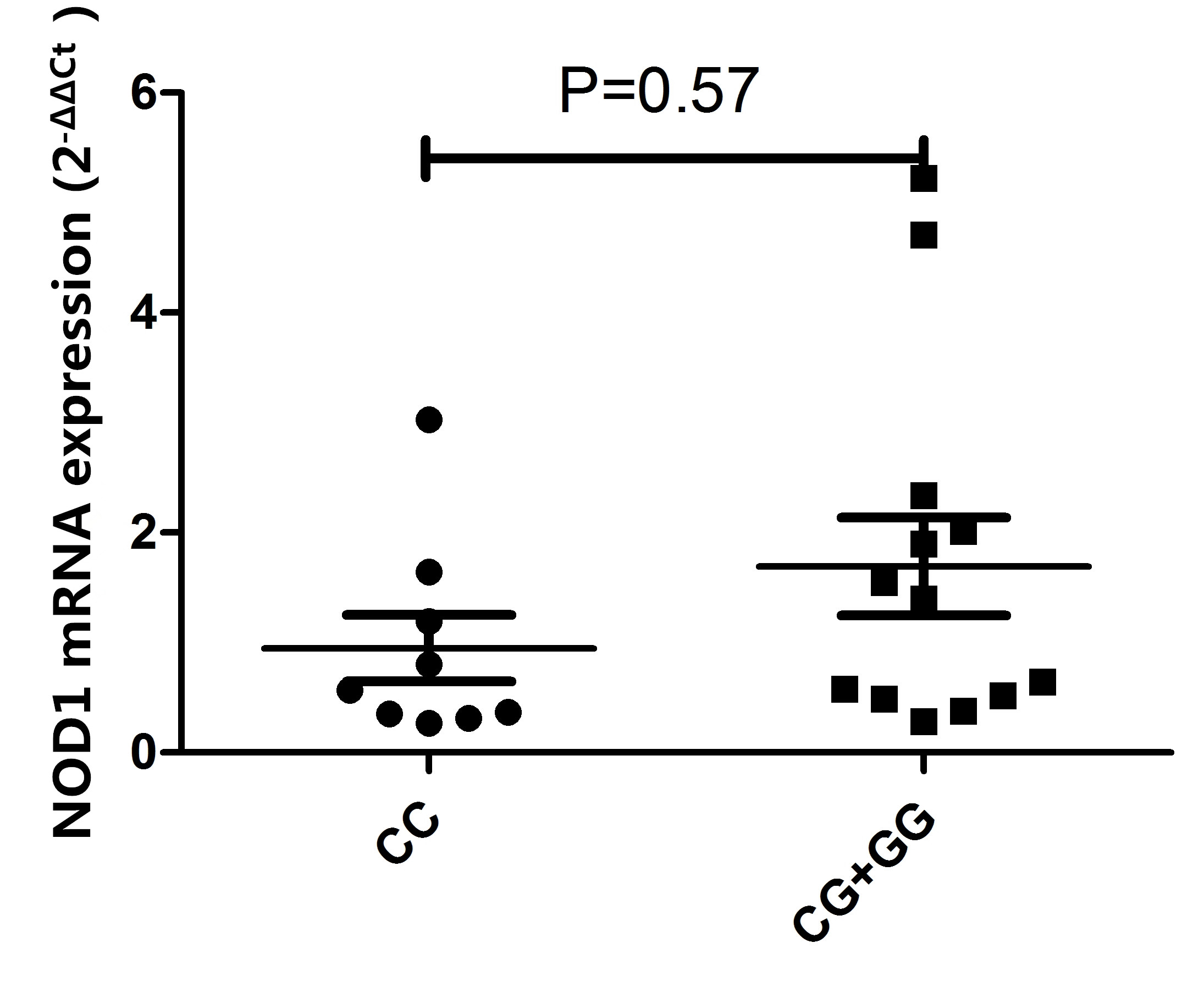
**

**Supplementary Figure S4.** The influence of various rs2075818 genotypes on

CIITA mRNA expression by PBMCs after stimulation with LPS.

**
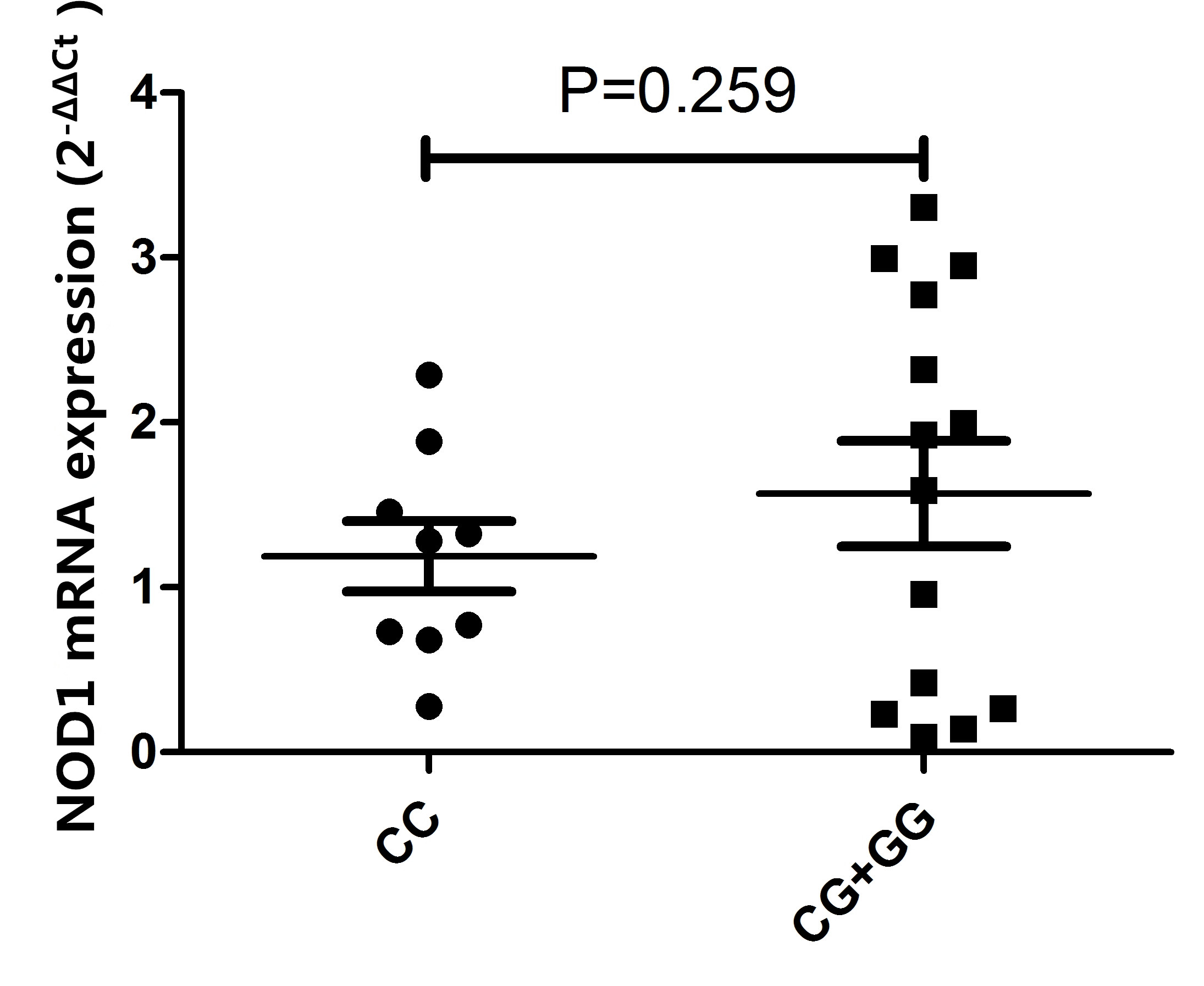
**

**Supplementary Figure S5.** The influence of various rs2075818 genotypes on

CIITA mRNA expression after stimulation of PBMCs with anti-CD3/CD28

antibodies.

**
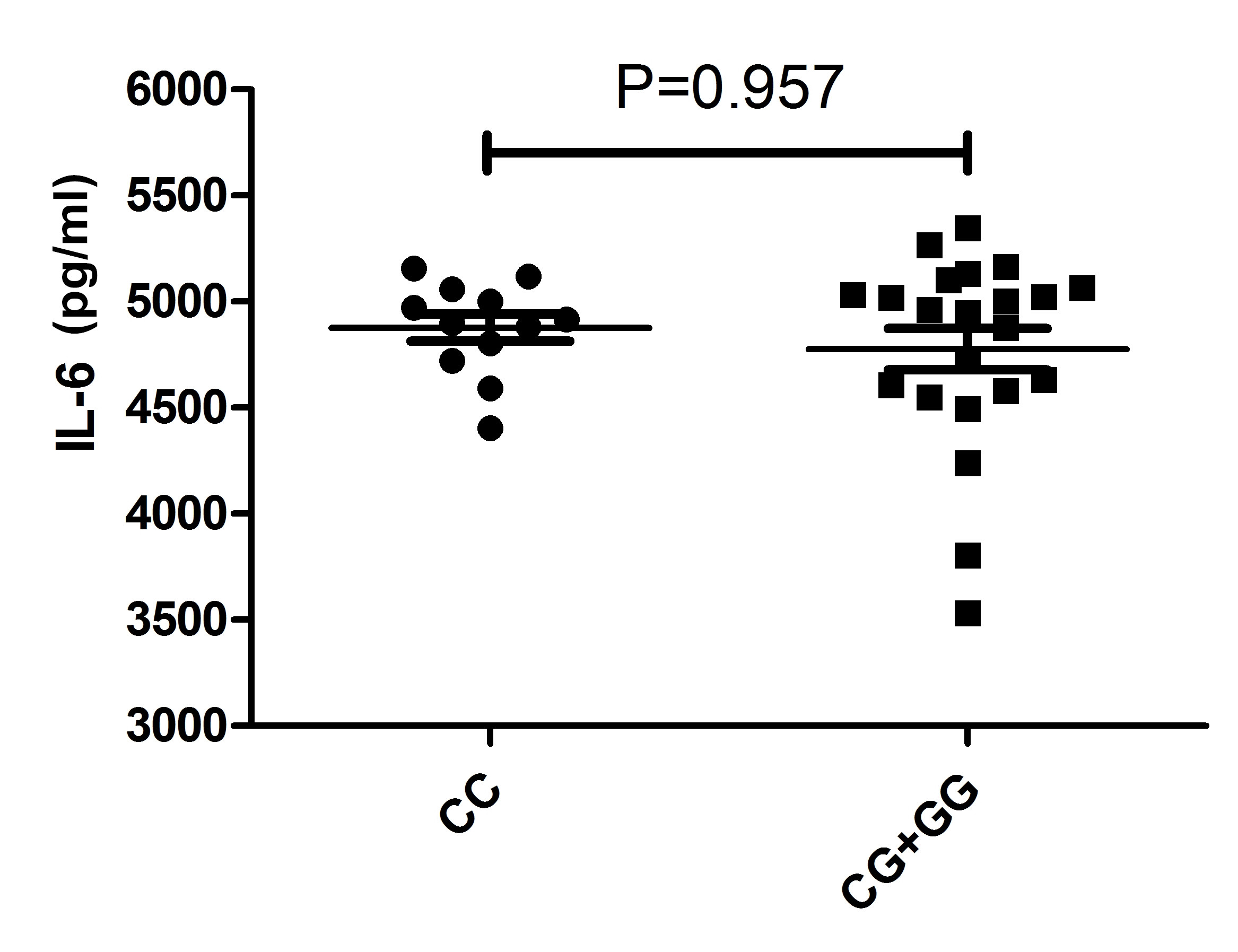
**

**Supplementary Figure S6.** The influence of various rs12932187 genotypes on

secretion of IL-6 by PBMCs after stimulation with LPS.

**
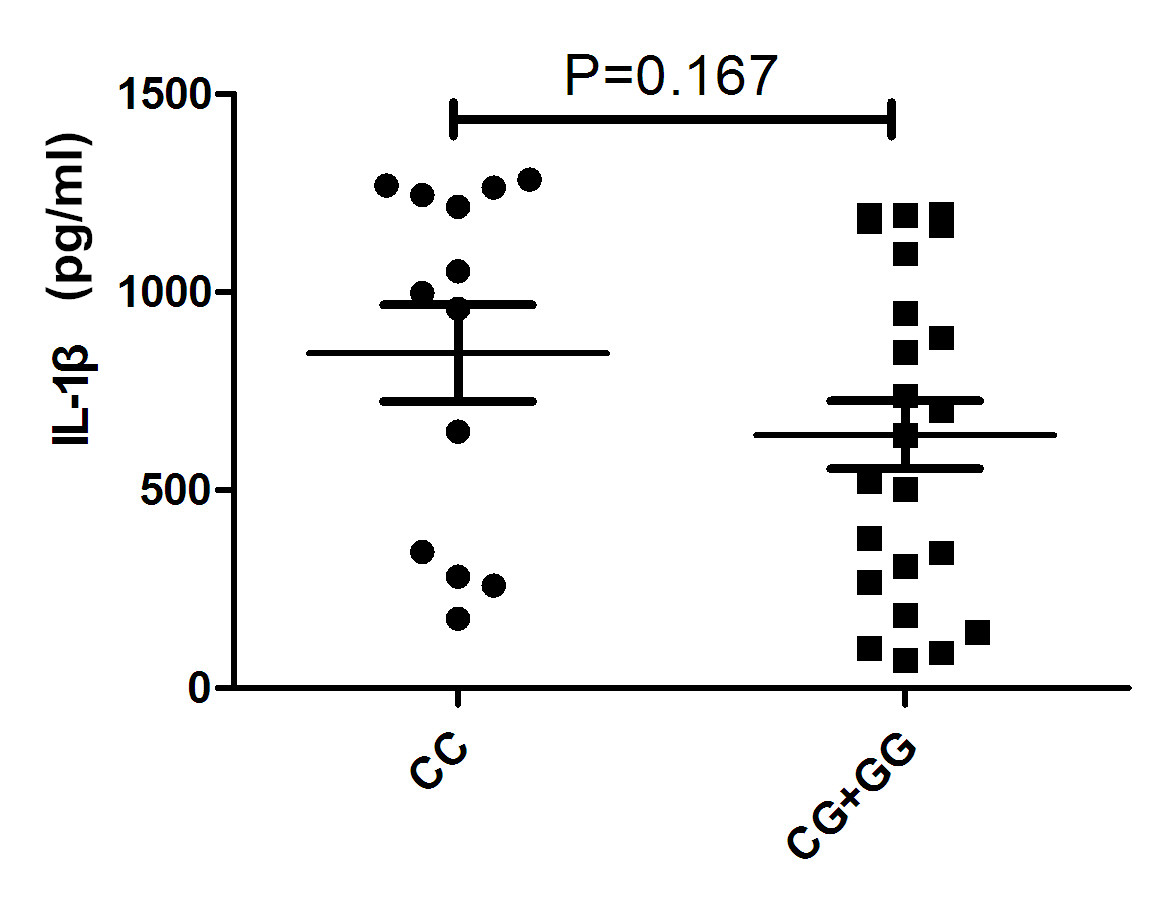
**

**Supplementary Figure S7.** The influence of various rs12932187 genotypes on

secretion of IL-1β by PBMCs after stimulation with LPS.

**
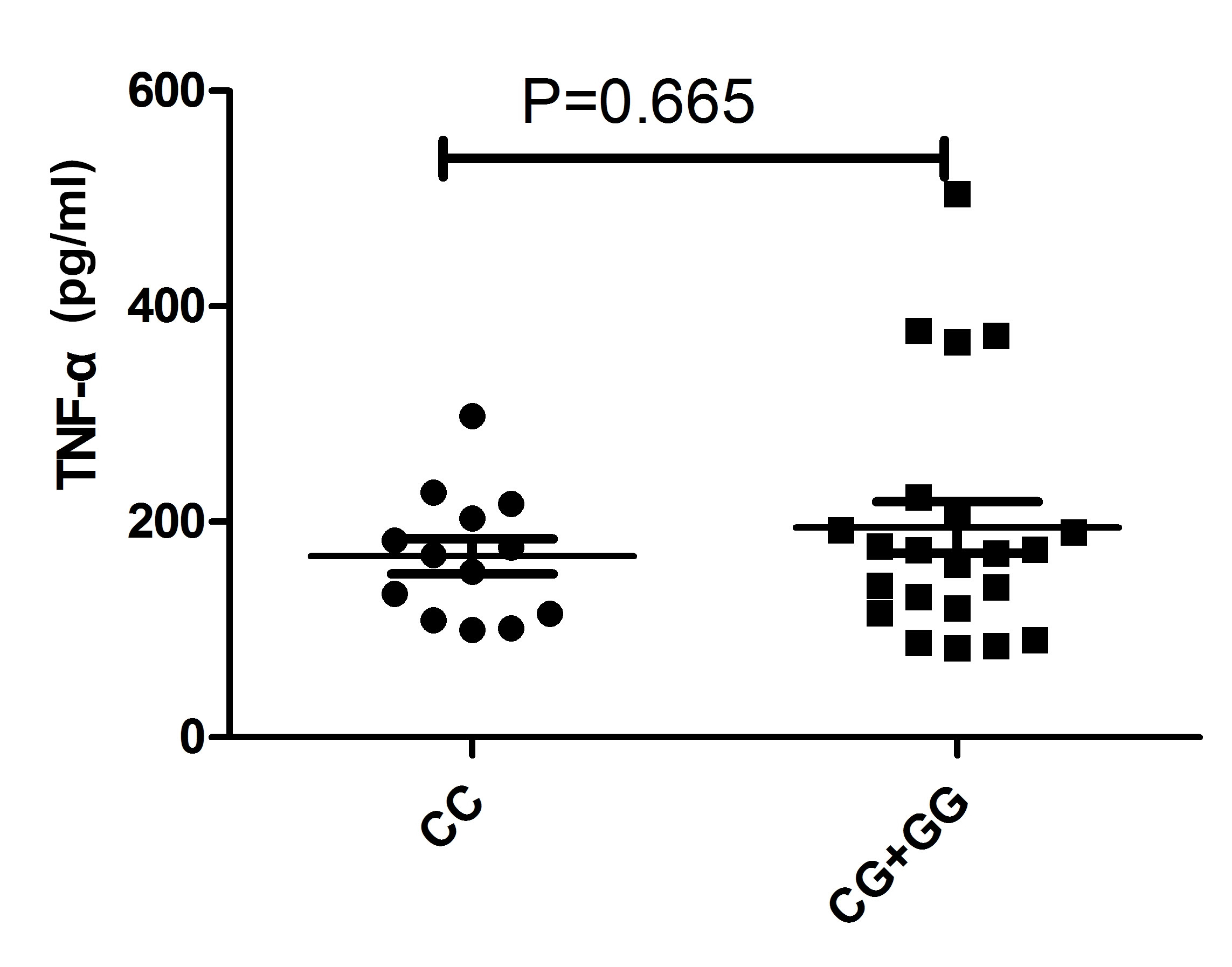
**

**Supplementary Figure S8.** The influence of various rs12932187 genotypes on

secretion of TNF-α by PBMCs after stimulation with LPS.

**
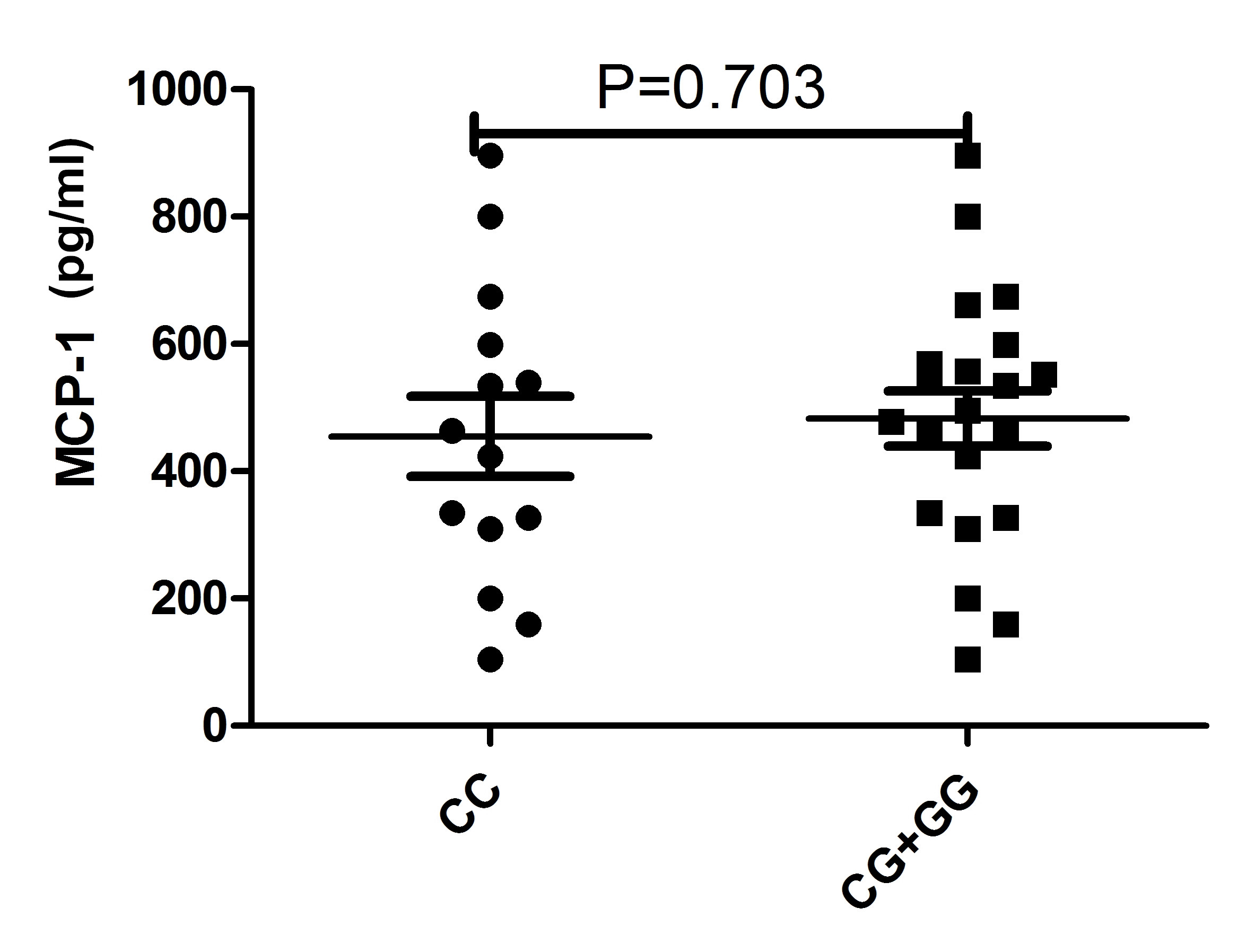
**

**Supplementary Figure S9.** The influence of various rs12932187 genotypes on

secretion of MCP-1 by PBMCs after stimulation with LPS.

**
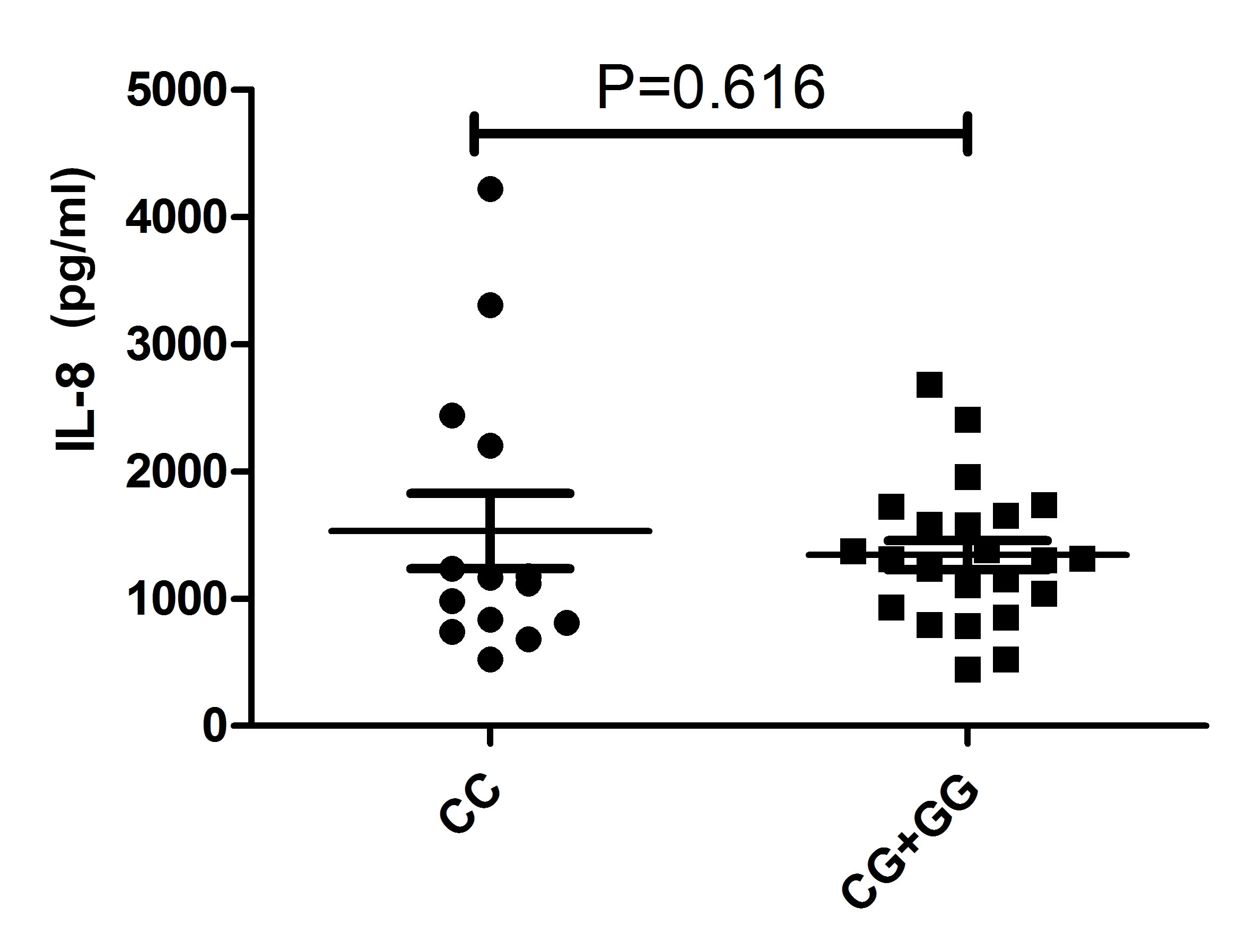
**

**Supplementary Figure S10.** The influence of various rs12932187 genotypes on

secretion of IL-8 by PBMCs after stimulation with LPS.
